# Perioperative Management of Ehlers-Danlos Type III Syndrome Associated With Postural Orthostatic Tachycardia in Patients Undergoing General Anesthesia

**DOI:** 10.7759/cureus.19311

**Published:** 2021-11-06

**Authors:** Andres Laserna, Mahd Nishtar, Courtney Vidovich, Zana Borovcanin

**Affiliations:** 1 Anesthesiology and Perioperative Medicine, University of Rochester School of Medicine and Dentistry, Rochester, USA

**Keywords:** ehlers-danlos syndrome, postural orthostatic tachycardia syndrome, general anesthesia, perioperative management, anesthesiology

## Abstract

Ehlers-Danlos syndrome (EDS) is an autosomal dominant inherited disorder of connective tissue with common clinical features of skin hyperelasticity, joint hypermobility, and easy bruising. Postural orthostatic tachycardia syndrome (POTS) refers to more than three months of a sustained increase in heart rate of more than 30 beats per minute and symptoms of orthostatic intolerance within 10 minutes of assuming a standing position without associated hypotension. These medical conditions can be associated with each other, potentially creating significant perioperative challenges. This paper describes two cases of young women with POTS and EDS hypermobility type (III) who presented for surgery under general anesthesia. The anesthesiologist performed an extensive preoperative evaluation, provided adequate preoperative hydration, ensured careful positioning during anesthetic induction, and avoided neck hyperextension during intubation. Gentle emergence and extubation were practiced with vigilance towards complications of cervical subluxation and airway mucosal injury. Robust communication between postoperative caregivers was prioritized. All these considerations facilitated the achievement of good outcomes. Here, a literature review and subsequent flow diagram of the anesthetic management and perioperative considerations for these patients is purposed.

## Introduction

Ehlers-Danlos syndrome (EDS) is a group of predominantly autosomal dominant inherited disorders of connective tissue, with common clinical features of skin hyperelasticity, joint hypermobility, and easy bruising [1]. It affects 1:10,000 to 1:25,000 individuals, resulting in at least 20,000-50,000 affected patients in North America [1]. Villefranche nosology describes at least six variants of EDS, which differ from each other according to the tissues involved and clinical manifestations. One of these variants, EDS hypermobility type (EDS III), comprises 30% of EDS cases [2,3]. The clinical features of EDS III consist of two major criteria (skin hyperextensibility and generalized joint hypermobility) as well as three minor criteria (recurring joint dislocations, chronic joint pain, and positive family history) [2]. Further, EDS III is uniquely associated with postural orthostatic tachycardia syndrome (POTS) [4].

POTS is a syndrome characterized by at least three months of a sustained increase in heart rate of 30 beats per minute or more within 10 minutes of assuming a standing position without associated hypotension or symptoms of orthostatic intolerance, such as lightheadedness, dizziness, and presyncopal episodes [5,6]. Patients with POTS are predominantly female (87%), with a mean age of 30 years [6]. Although the association of concurrent EDS and POTS has been described, its incidence has not been investigated extensively, and the explanation for their association is not completely understood [5]. Theories suggest that in patients with POTS, the inability of the cardiovascular and neural systems to work together to facilitate blood supply in the direction against gravity might be potentiated by the altered structural and functional properties of the arterial wall and heart in patients with EDS [7]. However, in a small study, arterial stiffness and cardiac profiles of patients with EDS III and POTS were comparable to those of the control group [5].

Ultimately, the perioperative management of patients with these conditions requires a complete understanding of their physical limitations and the resultant challenges for anesthesiologists. Several case reports and literature reviews have described the anesthetic considerations for patients with EDS or POTS as independent entities or jointly through regional anesthesia [8,9]. In reviewing the literature, no prior reports have described general anesthetic management for patients with concurrent EDS and POTS. Here, the authors present two such cases. Informed consent was obtained for the presentation of these cases, and through a literature review, several perioperative considerations for these patients were identified. Furthermore, a management flow diagram was developed to facilitate an anesthetic management plan.

## Case presentation

Case 1

A 26-year-old female with EDS III, POTS, anxiety, asthma, anemia, and chronic fatigue syndrome presented for laparoscopic bilateral ovarian cystectomy and bilateral partial salpingectomy. Prior to surgery, the patient was assessed at a center for perioperative medicine, and an extensive evaluation was performed. She had an unremarkable echocardiogram with normal aortic root seven years prior. The patient reported a history of syncopal episodes associated with POTS, which required mediport placement at age 14 for infusions of normal saline for a total of nine years due to insufficient oral intake in the setting of esophageal dysmotility, a known risk factor for aspiration. 

At the time of the surgery, her symptoms had been managed with adequate oral hydration and sodium tablets. She reported a history of delayed emergence with general anesthesia during mediport placement when she was 14 but otherwise had no previous anesthetic complications. On the day of surgery, the patient, accompanied by her mother, was evaluated (Ht 1.70 m, Wt: 71 kg, BMI: 24 Kg/m2, BP: 105/63 mmHg, HR: 70-80s, EKG normal sinus rhythm, clear breath sounds, normal airway, Mallampati II). A thorough discussion regarding the anesthetic management and considerations related to her medical conditions was carried out. Given her joint laxity, the patient and her mother expressed concerns about intubation and head positioning during surgery, but they were reassured by the attending anesthesiologist. Intraoperatively, standard American Society of Anesthesiologists (ASA) monitors were applied and a surgical timeout was performed, detailing the patient's unique risk factors, including increased risk for aspiration, joint injuries, and hemodynamic derangements. After adequate preoxygenation, she was induced with fentanyl 100 mcg, lidocaine 60 mg, propofol 100 mg, and rocuronium 50 mg. Her eyelids were closed and gently covered with plastic tape to avoid eye pressure. After we confirmed that the patient was paralyzed with no twitch response, we intubated her using a video laryngoscope (Grade I view) with her head placed in a neutral position to avoid neck hyperextension and anterior traction.

All operating room staff, including the surgical team, were notified about her hypermobility syndrome, and she was placed in a lithotomy position with slow and gentle movements and foam cushions to support her joints. She was maintained under general anesthesia with sevoflurane 0.9 MAC under volume control AutoFlow (VC-AF) ventilation. Vital signs during the surgery were stable with a mean arterial pressure (MAP) of 62 mmHg (range 50 mmHg-117 mmHg). She received and adequately responded to ephedrine 5 mg intravenously on three occasions in the setting of abdominal insufflation when the MAP was below 60 mmHg and heart rate (HR) was between 50-65 bpm. During the three-hour-long procedure, no surgical complications were noted, and the patient received a total of 1.6 L of Lactated Ringer’s solution, had an estimated blood loss of 50 ml, and urine output of 250 ml. After a gradual and gentle emergence with minimal coughing and bucking, she was extubated without complications and transferred to the PACU for complete recovery. The nursing staff was notified about the special considerations regarding changes in positions and possible vital sign fluctuations that could be observed, including joint laxity and unexplained tachycardia. She had no complications and was discharged home. One week later, she followed up with her gynecologist, who reported that her surgery had gone well without complications. 

Case 2

A 20-year-old female with a past medical history of Graves’ disease, EDS III, POTS, migraine headaches, systemic mastocytosis, depression, and anxiety presented to the hospital for thyroidectomy. She was evaluated at a center for perioperative medicine, where she explained that her EDS III was characterized by fatigue, constipation, dizziness, joint hypermobility, right knee subluxation, and chronic generalized musculoskeletal pain. She denied previous hospitalizations or history of anesthetic complications, and her medical regimen included propranolol 10 mg nightly, amitriptyline 20 mg nightly, meloxicam 7.5 mg as needed, fludrocortisone 0.15 mg daily, ivabradine 0.5 mg twice daily, and salt tablets 20 mEq thrice a week.

Her POTS, diagnosed at the age of sixteen via a tilt table test, was associated with postural presyncopal episodes, dizziness, and lightheadedness with prolonged standing, and intermittent palpitations with moderate activity. Her symptoms were exacerbated during the winter months and were managed with intravenous infusions of normal saline, salt tablets (20 mEq thrice a week), fludrocortisone (0.15 mg daily), ivabradine (0.5 mg twice daily), and compression stockings. Otherwise, her symptoms were managed conservatively with liberalized salt and fluid intake as well as a low-level aerobic activity. She did not have a previous echocardiogram but had frequent follow-up visits with a cardiologist who had cleared her for surgery and determined that she did not need further cardiac workup. Her preoperative EKG was read as normal sinus rhythm at 78 beats per minute. On the day of the surgery, she was accompanied by her mother, who gave the providers a folder with perioperative recommendations for patients with EDS and POTS. She was very concerned about the procedure and the risk of joint luxation and was reassured by the team regarding the anesthetic plan once the special considerations were explained. Her exam was unremarkable (Ht 1.55 m, Wt: 48.1 kg, BMI: 20 Kg/m2, BP: 108/76 mmHg, HR: 70-80s, normal sinus rhythm, clear breath sounds, normal airway), and she received 1L of Lactated Ringer’s in the preoperative period. In the operating room during the surgical timeout, the entire team was made aware of the considerations for the management of the patient.

After she was placed under standard ASA monitors and received adequate preoxygenation, the patient was induced with fentanyl 100 mcg, lidocaine 50 mg, propofol 100 mg, and succinylcholine 100 mg. Her eyelids were closed and gently covered with plastic tape to avoid eye pressure. After the patient was mask ventilated, she was subsequently intubated with a neural integrity monitor (NIM) endotracheal tube (ETT) using a video laryngoscope (Grade I view), with her head placed in a slightly extended position, to provide adequate visualization of the surgical field. Given the patient's history of presyncopal episodes as well as chronic fludrocortisone therapy, a postinduction arterial line was placed using an ultrasound-guided technique to allow for beat-to-beat hemodynamic monitoring. Anesthesia was maintained with sevoflurane 0.8-1.0 MAC under volume control ventilation. Vital signs during the surgery were stable, with a mean MAP of 72 mmHg (range 57-107 mmHg) with no vasopressor medications required. After a two-hour-long procedure, the patient received a total of 1.4 L Lactated Ringer’s and 1 L Plasmalyte with 10 ml of estimated blood loss. She was transferred to the PACU, recovered appropriately over two hours, and was discharged home. Two weeks later, she visited the outpatient surgical oncology team for post-operative follow-up of her total thyroidectomy and was noted to have recovered very nicely from surgery.

## Discussion

**Figure 1:**
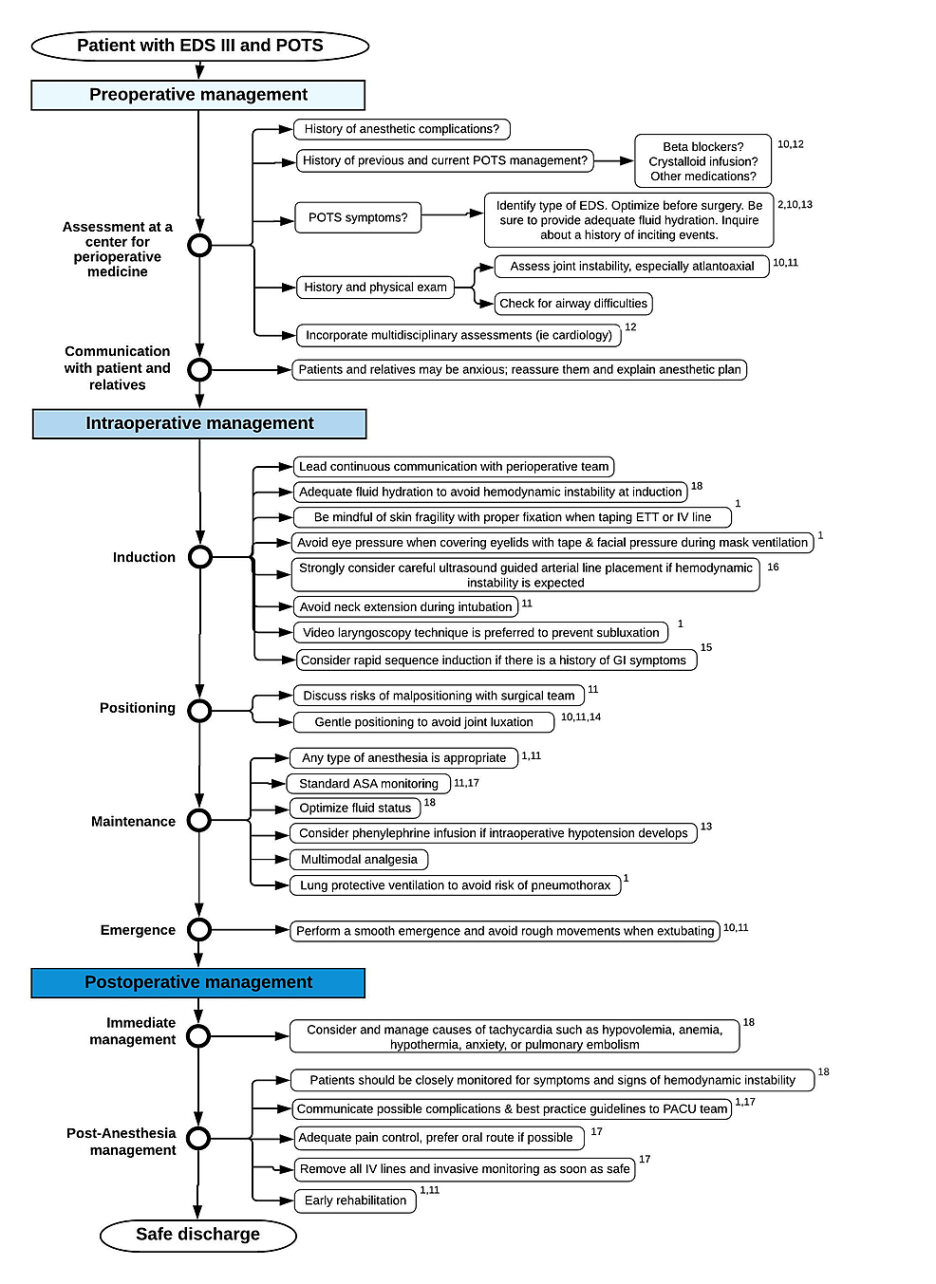
Perioperative flow diagram for the management of patients with EDS III and POTS POTS: Postural Orthostatic Tachycardia Syndrome, EDS III: Ehlers-Danlos Syndrome Type III, ASA: American Society of Anesthesiologists, PACU: Post-Anesthesia Care Unit, ETT: endotracheal tube, IV: Intravenous

We present the successful anesthetic management of two patients with EDS III associated with POTS undergoing general anesthesia. Both patients had an extensive preoperative evaluation, received adequate preoperative hydration, were carefully positioned intraoperatively, and underwent anesthetic procedures with the utmost diligence. All these considerations facilitated good outcomes and adequate recovery. After debriefing and discussing the cases, we conducted a comprehensive literature review to develop the first publication, to our knowledge, that comprehensively describes evidence-based perioperative management for patients with EDS III associated with POTS undergoing general anesthesia. The flow diagram presented in this review can contribute to the perioperative planning for these patients (Figure 1).

Our literature review used Medline and Embase from inception to March 1, 2021, with the following terms: Ehlers-Danlos syndrome, Ehlers-Danlos syndrome type III, postural orthostatic tachycardia syndrome, and anesthesia, perioperative care, perioperative period, perioperative medicine, preoperative, intraoperative, and postoperative. Our inclusion criteria were articles in the English or Spanish language in which the perioperative care of adult patients with EDS III or POTS undergoing surgical procedures under general anesthesia was discussed. 

Preoperative management

Preoperatively, our cases and subsequent review shed light on the importance of a multidisciplinary evaluation to optimize the medical conditions of these patients. Beginning with EDS III, a thorough medical history should be gathered to assess for common conditions, including cardiac abnormalities and joint dislocations, joint injuries, and atlantoaxial instability [10,11]. For cardiac abnormalities, anesthesiologists should obtain and review a detailed cardiac workup before surgery. If warranted, a cardiologist may further evaluate the patient for aortic aneurysms, valvular heart disease, and necessary medications such as beta-blockers for surgical clearance [12]. In our cases, both patients were assessed at a center for perioperative medicine with input from their respective outpatient cardiologists. 

Similarly, for POTS, a multidisciplinary approach is imperative to mitigate the stress of surgery with appropriate medication and fluid optimization [2]. NPO guidelines result in a tendency for impaired sympathetic vasoconstriction and volume dysregulation. Therefore, it is advised that preoperative intravenous hydration be given before the administration of sedative or anesthetic medications [13]. Beta-blockers should be continued, and in those taking fludrocortisone therapy, it is reasonable to continue the medication in the perioperative period, while monitoring for signs of adrenal insufficiency. Additionally, in highly symptomatic patients with significant orthostatic blood pressure changes, a pre-induction arterial line should be considered, and midodrine can be given 30 minutes before surgical intervention [13].

Intraoperative management

Intraoperatively, gentle patient positioning and careful intubation are crucial factors due to innate tissue fragility and the propensity for pain, joint dislocation, cervical spine instability, bleeding, and hematoma formation in EDS III [10,11,14]. When positioning, gentle movements, avoidance of the lithotomy position, as able, and adequate protection of pressure areas are encouraged with attention to any prior history of joint pain and dislocation [11]. During intubation, standard induction is appropriate, although a rapid sequence induction should be considered for patients at higher risk of aspiration. One retrospective study found that both EDS III and POTS are independent risk factors for gastrointestinal (GI) symptoms, including GI dysmotility and gastroesophageal reflux disease [15]. As such, those patients presenting with GI symptoms should be considered for rapid sequence induction. Further, video laryngoscopy is preferred to avoid injury resulting in bleeding with subsequent airway compromise, a technique used in both of our patients [1]. Additionally, gentle temporomandibular joint and minimal cervical spine movements are best, and awake fiberoptic intubation may be considered [11]. When mask ventilating, pressure applied should be monitored to avoid facial bruising, and low airway pressures should be maintained due to the higher risk of pneumothoraces [1]. 

A second intraoperative concern is invasive hemodynamic monitoring for continuous blood pressure measurement. Although invasive monitoring places patients with EDS III at risk for vascular damage, it may be valuable in the management of POTS given the potential for rapid hemodynamic changes, as seen in both of our patients [1,16] Further, if hemodynamic instability occurs without invasive monitoring, it would be difficult and potentially traumatic to establish arterial access quickly, even with ultrasound guidance [4]. For our cases, one was monitored with a non-invasive cuff and the other had a post-induction ultrasound-guided arterial line. Additionally, a preoperative EKG should be considered to evaluate for underlying cardiac arrhythmias [17].

A third intraoperative consideration is the importance of fluid resuscitation and the use of vasopressors. In a retrospective study, three out of 13 patients with POTS had prolonged intraoperative hypotension [18]. As such, fluid resuscitation with balanced crystalloid solutions is recommended preoperatively to improve preload in patients who experience hypotension [18]. We implemented this for one of our patients. Further, in patients with persistent hypotension despite fluid resuscitation, phenylephrine should be used as a first-line medication due to its selective alpha-1 adrenergic activity, since ephedrine, with its beta-1 effect, can worsen tachycardia. Vasopressin can be also used as an alternative vasopressor [13]. In one of our cases, the patient required boluses of ephedrine on three occasions when her heart rate was low. She had an adequate hemodynamic response without excessive tachycardia.

Postoperative management

Positioning recommendations continue postoperatively as well as avoidance of skin trauma from subcutaneous and intramuscular injections, with quick removal of intravenous lines or invasive monitoring devices [16]. Patients with EDS III should be monitored for high risk of surgical emphysema secondary to accidental tracheal puncture and pleural effusion or tamponade from central venous catheters [16]. Pain management can also be challenging when relying on regional analgesia, as it has been found that patients with EDS may be resistant to local anesthetics [11]. Further, patients with POTS should be closely monitored with a routine evaluation of orthostatic vitals. There is no need for hospitalization, intensive care unit admission, or extensive postoperative testing (i.e., EKG) on a routine basis [17]. Finally, early mobilization postoperatively is essential to prevent deconditioning [1,11].

Communication was critical in both of our cases. During the entire perioperative period, the anesthesiology team led communication with surgeons, nursing staff, and the family. Communication is vital in risk reduction, especially for these patients, and likely contributed to the successful perioperative outcomes of our patients.

## Conclusions

EDS III can be associated with POTS, and these patients will require a multidisciplinary preoperative assessment with the operating room staff and post-anesthesia care nurse's active participation to assure good outcomes and patient satisfaction. Anesthesiologists should be aware of their critical role in the care of these patients. The flow diagram presented here should become a useful tool for anesthetic management and perioperative planning.
